# 4-Acetyl-1*H*-pyrrole-2-carbaldehyde

**DOI:** 10.1107/S1600536812039153

**Published:** 2012-09-19

**Authors:** Tao Sun, Jia-Wei Xie, Ren-Ying Zhao, Ai-Guo Zhu, Yan-Qing Ge

**Affiliations:** a Taishan Medical University, Tai’an 271016, People’s Republic of China

## Abstract

The title compound, C_7_H_7_NO_2_, was synthesized *via* a one-pot Vilsmeier–Haack and subsequent Friedel–Crafts reaction. The pyrazole ring makes dihedral angles of 4.50 (9) and 2.06 (8)°, respectively, with the aldehyde and acetyl groups. In the crystal, classical N—H⋯O hydrogen bonds and weak C—H⋯O inter­actions assemble the mol­ecules into a chain along the *b* axis.

## Related literature

For the synthetic procedure, see: Ge *et al.* (2009 ▶). For related structures, see: Ge *et al.* (2011 ▶); Hao *et al.* (2012 ▶).
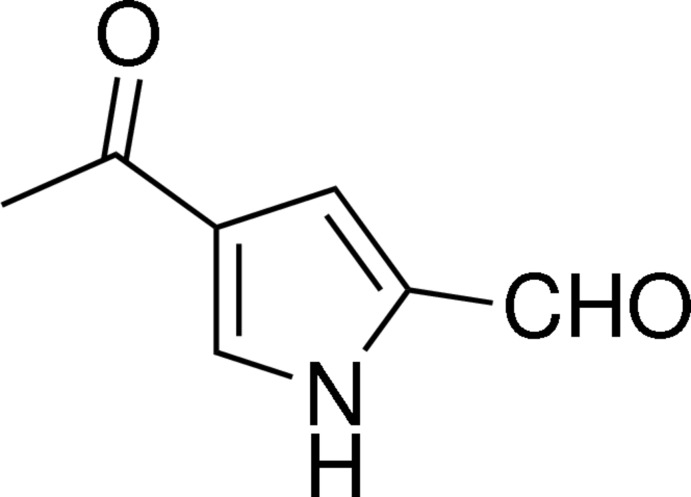


## Experimental

### 

#### Crystal data

C_7_H_7_NO_2_
*M* *_r_* = 137.14
Monoclinic, 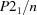

*a* = 3.811 (5) Å
*b* = 13.219 (5) Å
*c* = 13.167 (5) Å
β = 95.602 (5)°
*V* = 660.2 (9) Å^3^
*Z* = 4
Mo *K*α radiation
μ = 0.10 mm^−1^
*T* = 293 K
0.15 × 0.13 × 0.10 mm

#### Data collection

Bruker APEXII CCD area-detector diffractometer
Absorption correction: multi-scan (*SADABS*; Bruker, 2005 ▶) *T*_min_ = 0.541, *T*_max_ = 0.556
3752 measured reflections
1348 independent reflections
1010 reflections with *I* > 2σ(*I*)
*R*_int_ = 0.056

#### Refinement

*R*[*F* ^2^ > 2σ(*F* ^2^)] = 0.042
*wR*(*F* ^2^) = 0.128
*S* = 1.04
1348 reflections
93 parameters
H-atom parameters constrained
Δρ_max_ = 0.20 e Å^−3^
Δρ_min_ = −0.14 e Å^−3^

### 

Data collection: *APEX2* (Bruker, 2005 ▶); cell refinement: *SAINT* (Bruker, 2005 ▶); data reduction: *SAINT*; program(s) used to solve structure: *SIR97* (Altomare *et al.*, 1999 ▶); program(s) used to refine structure: *SHELXL97* (Sheldrick, 2008 ▶); molecular graphics: *XP* in *SHELXTL* (Sheldrick, 2008 ▶); software used to prepare material for publication: *WinGX* (Farrugia, 1999 ▶).

## Supplementary Material

Crystal structure: contains datablock(s) I, global. DOI: 10.1107/S1600536812039153/im2396sup1.cif

Structure factors: contains datablock(s) I. DOI: 10.1107/S1600536812039153/im2396Isup2.hkl

Supplementary material file. DOI: 10.1107/S1600536812039153/im2396Isup3.cml

Additional supplementary materials:  crystallographic information; 3D view; checkCIF report

## Figures and Tables

**Table 1:** Hydrogen-bond geometry (Å, °)

*D*—H⋯*A*	*D*—H	H⋯*A*	*D*⋯*A*	*D*—H⋯*A*
N1—H1*A*⋯O2^i^	0.86	2.11	2.876 (2)	148
C7—H7*A*⋯O1^ii^	0.96	2.54	3.453 (5)	159

Symmetry codes: (i) 
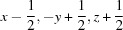
; (ii) 
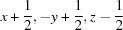
.

**Fig. 1.**
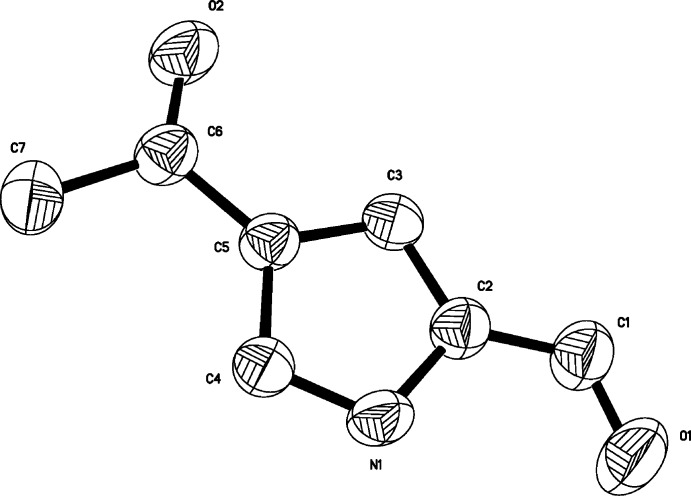
The molecular structure of compound, showing displacement ellipsoids drawn at the 30% probability level.

**Fig. 2.**
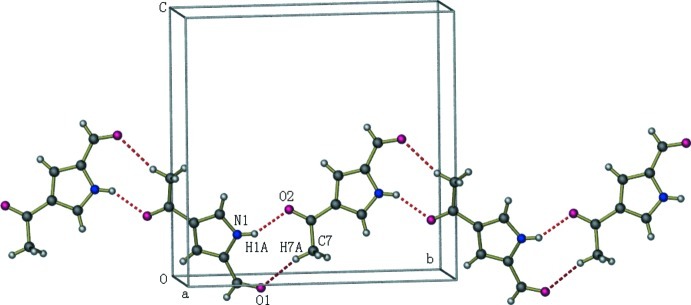
The one-dimensional infinite chain structure, which is formed by intermolecular N—H···O hydrogen bonding and weak C—H···O interactions.

## supplementary crystallographic information

### Comment

Synthesis of nitrogen-containing heterocyclic compounds has been a subject of great interest due to the wide application in agrochemical and pharmaceutical fields (Ge *et al.*; 2009, 2011). Some pyrrole derivatives which belong to this category exhibit interesting biological properties, such as anti-bacterial, anti-inflammatory, anti-oxidant, anti-tumor, anti-fungal, and immune suppressant activities. The title pyrazole (I) (Fig. 1) was synthesized in order to study its biological properties. (I) was screened for anticancer activities and found to be inactive. We report here the crystal structure of the title compound. In the title compound, C_7_H_7_NO_2_, the pyrazole ring makes dihedral angles of 4.50 (9)° and 2.06 (8)°, respectively, with the aldehyde group and acetyl group. The crystal structure is determined by classical intermolecular N—H···O (H1A···O2 = 2.11 Å) hydrogen bonding and weak C—H···O (H7A···O1 = 2.54 Å) interactions, which assemble the molecules into a one-dimensional chain structure.

### Experimental

A solution of dimethylformamide (5.5 mmol) in 1,2-dichloroethane (10 ml) in a 3-necked flask was cooled in an ice bath. To the stirred and cooled solution was added a solution of oxalyl chloride (5.5 mmol) in 1,2-dichloroethane (10 ml) over a period of 10 min. The suspension of white solid was then allowed to stir at room temperature for 15 min. The suspension was cooled in ice and a solution of pyrrole (5 mmol) in 1.2-dichloroethane (10 ml) was added over 10 min. The light orange solution obtained was allowed to stir 15 min at room temperature.To this was added aluminium chloride (11 mmol), followed by acetyl chloride (5 mmol), rapidly and at room temperature. The mixture was stirred for 3 h. The mixture was then poured onto about 50 ml of ice and water, 50% aqueous sodium hydroxide (4 ml) was added, and the mixture was stirred rapidly for about 10 min. The mixture was than made slightly acidic with concentrated hydrochloric acid and the organic and aqueous layers were separated. The aqueous layer was extracted with ethyl ether. The organic extracts were washed with water, dried, filtered and concentrated. The final product was isolated by column chromatography on silica gel (yield 76%). Crystals of (I) suitable for X-ray diffraction were obtained by allowing a refluxed solution of the product in ethyl acetate (0.10 *M*) to cool slowly to room temperature (without temperature control) and allowing the solvent to evaporate for 3 d.

### Refinement

All H atoms were placed in geometrically calculated positions and refined using a riding model with C—H = 0.93 Å (for aldehyde group and pyrrole ring), C—H = 0.96 Å (for CH_3_) and N—H = 0.86 Å with U_iso_(H) = 1.2 U_eq_(C,N) for the aldehyde group and pyrrole hydrogen atoms and U_iso_(H) = 1.5 U_eq_(C) for CH_3_.

### Crystal data

**Table:**

C_7_H_7_NO_2_	*F*(000) = 288
*M**_r_* = 137.14	*D*_x_ = 1.380 Mg m^−^^3^
Monoclinic, *P*2_1_/*n*	Mo *K*α radiation, λ = 0.71069 Å
Hall symbol: -P 2yn	Cell parameters from 1324 reflections
*a* = 3.811 (5) Å	θ = 3.1–25.7°
*b* = 13.219 (5) Å	µ = 0.10 mm^−^^1^
*c* = 13.167 (5) Å	*T* = 293 K
β = 95.602 (5)°	Block, colorless
*V* = 660.2 (9) Å^3^	0.15 × 0.13 × 0.10 mm
*Z* = 4	

### Data collection

**Table:**

Bruker APEXII CCD area-detector diffractometer	1348 independent reflections
Radiation source: fine-focus sealed tube	1010 reflections with *I* > 2σ(*I*)
Graphite monochromator	*R*_int_ = 0.056
phi and ω scans	θ_max_ = 26.4°, θ_min_ = 2.2°
Absorption correction: multi-scan (*SADABS*; Bruker, 2005)	*h* = −4→4
*T*_min_ = 0.541, *T*_max_ = 0.556	*k* = −15→16
3752 measured reflections	*l* = −12→16

### Refinement

**Table:**

Refinement on *F*^2^	Secondary atom site location: difference Fourier map
Least-squares matrix: full	Hydrogen site location: inferred from neighbouring sites
*R*[*F*^2^ > 2σ(*F*^2^)] = 0.042	H-atom parameters constrained
*wR*(*F*^2^) = 0.128	*w* = 1/[σ^2^(*F*_o_^2^) + (0.0703*P*)^2^] where *P* = (*F*_o_^2^ + 2*F*_c_^2^)/3
*S* = 1.04	(Δ/σ)_max_ < 0.001
1348 reflections	Δρ_max_ = 0.20 e Å^−^^3^
93 parameters	Δρ_min_ = −0.14 e Å^−^^3^
0 restraints	Extinction correction: *SHELXL97* (Sheldrick, 2008), Fc^*^=kFc[1+0.001xFc^2^λ^3^/sin(2θ)]^-1/4^
Primary atom site location: structure-invariant direct methods	Extinction coefficient: 0.031 (9)

### Special details

**Table:**

Geometry. All e.s.d.'s (except the e.s.d. in the dihedral angle between two l.s. planes) are estimated using the full covariance matrix. The cell e.s.d.'s are taken into account individually in the estimation of e.s.d.'s in distances, angles and torsion angles; correlations between e.s.d.'s in cell parameters are only used when they are defined by crystal symmetry. An approximate (isotropic) treatment of cell e.s.d.'s is used for estimating e.s.d.'s involving l.s. planes.
Refinement. Refinement of *F*^2^ against ALL reflections. The weighted *R*-factor *wR* and goodness of fit *S* are based on *F*^2^, conventional *R*-factors *R* are based on *F*, with *F* set to zero for negative *F*^2^. The threshold expression of *F*^2^ > σ(*F*^2^) is used only for calculating *R*-factors(gt) *etc*. and is not relevant to the choice of reflections for refinement. *R*-factors based on *F*^2^ are statistically about twice as large as those based on *F*, and *R*- factors based on ALL data will be even larger.

### Fractional atomic coordinates and isotropic or equivalent isotropic displacement parameters (Å^2^)

**Table:**

	*x*	*y*	*z*	*U*_iso_*/*U*_eq_	
N1	0.4828 (4)	0.16971 (9)	0.21257 (10)	0.0472 (4)	
H1A	0.4131	0.1739	0.2726	0.057*	
O2	0.7983 (4)	0.23425 (9)	−0.10389 (9)	0.0634 (4)	
C4	0.5018 (4)	0.24663 (12)	0.14876 (11)	0.0448 (4)	
H4	0.4423	0.3133	0.1619	0.054*	
C6	0.6799 (4)	0.27169 (12)	−0.02967 (12)	0.0459 (4)	
C2	0.5910 (4)	0.08234 (11)	0.16858 (11)	0.0452 (4)	
O1	0.4742 (4)	−0.03023 (10)	0.29831 (10)	0.0777 (5)	
C3	0.6811 (4)	0.10776 (11)	0.07349 (12)	0.0437 (4)	
H3	0.7652	0.0638	0.0264	0.052*	
C5	0.6236 (4)	0.21205 (11)	0.05991 (11)	0.0413 (4)	
C7	0.5885 (6)	0.38131 (13)	−0.02795 (14)	0.0606 (5)	
H7A	0.6548	0.4132	−0.0887	0.091*	
H7B	0.7126	0.4126	0.0308	0.091*	
H7C	0.3392	0.3887	−0.0248	0.091*	
C1	0.5825 (5)	−0.01457 (13)	0.21628 (14)	0.0582 (5)	
H1	0.6661	−0.0696	0.1819	0.070*	

### Atomic displacement parameters (Å^2^)

**Table:**

	*U*^11^	*U*^22^	*U*^33^	*U*^12^	*U*^13^	*U*^23^
N1	0.0623 (9)	0.0475 (8)	0.0331 (7)	−0.0014 (6)	0.0119 (6)	−0.0040 (6)
O2	0.0926 (10)	0.0596 (8)	0.0412 (7)	−0.0007 (6)	0.0225 (6)	−0.0005 (6)
C4	0.0576 (10)	0.0385 (8)	0.0390 (9)	0.0007 (7)	0.0082 (7)	−0.0040 (7)
C6	0.0535 (9)	0.0477 (9)	0.0366 (9)	−0.0042 (7)	0.0046 (7)	−0.0013 (7)
C2	0.0538 (10)	0.0432 (9)	0.0383 (9)	−0.0001 (7)	0.0026 (7)	−0.0017 (7)
O1	0.1213 (12)	0.0589 (9)	0.0546 (9)	−0.0035 (7)	0.0173 (8)	0.0133 (7)
C3	0.0503 (9)	0.0423 (9)	0.0389 (9)	0.0017 (7)	0.0065 (7)	−0.0067 (7)
C5	0.0467 (9)	0.0420 (9)	0.0356 (8)	−0.0020 (6)	0.0053 (6)	−0.0024 (6)
C7	0.0764 (12)	0.0480 (10)	0.0585 (11)	0.0044 (9)	0.0112 (9)	0.0073 (8)
C1	0.0797 (13)	0.0467 (10)	0.0480 (10)	−0.0001 (8)	0.0052 (9)	0.0011 (8)

### Geometric parameters (Å, º)

**Table:**

N1—C4	1.325 (2)	C2—C1	1.429 (2)
N1—C2	1.373 (2)	O1—C1	1.211 (2)
N1—H1A	0.8600	C3—C5	1.405 (2)
O2—C6	1.2207 (18)	C3—H3	0.9300
C4—C5	1.378 (2)	C7—H7A	0.9600
C4—H4	0.9300	C7—H7B	0.9600
C6—C5	1.452 (2)	C7—H7C	0.9600
C6—C7	1.491 (2)	C1—H1	0.9300
C2—C3	1.372 (2)		
			
C4—N1—C2	109.91 (13)	C5—C3—H3	126.1
C4—N1—H1A	125.0	C4—C5—C3	106.19 (13)
C2—N1—H1A	125.0	C4—C5—C6	126.74 (14)
N1—C4—C5	109.11 (13)	C3—C5—C6	127.07 (13)
N1—C4—H4	125.4	C6—C7—H7A	109.5
C5—C4—H4	125.4	C6—C7—H7B	109.5
O2—C6—C5	121.72 (16)	H7A—C7—H7B	109.5
O2—C6—C7	120.76 (14)	C6—C7—H7C	109.5
C5—C6—C7	117.52 (14)	H7A—C7—H7C	109.5
C3—C2—N1	106.92 (14)	H7B—C7—H7C	109.5
C3—C2—C1	129.75 (15)	O1—C1—C2	124.74 (17)
N1—C2—C1	123.23 (15)	O1—C1—H1	117.6
C2—C3—C5	107.87 (13)	C2—C1—H1	117.6
C2—C3—H3	126.1		
			
C2—N1—C4—C5	0.08 (18)	C2—C3—C5—C6	−179.38 (15)
C4—N1—C2—C3	0.20 (18)	O2—C6—C5—C4	177.98 (16)
C4—N1—C2—C1	−176.51 (16)	C7—C6—C5—C4	−1.8 (2)
N1—C2—C3—C5	−0.40 (17)	O2—C6—C5—C3	−2.2 (2)
C1—C2—C3—C5	176.02 (16)	C7—C6—C5—C3	177.96 (15)
N1—C4—C5—C3	−0.33 (17)	C3—C2—C1—O1	−174.36 (18)
N1—C4—C5—C6	179.50 (14)	N1—C2—C1—O1	1.5 (3)
C2—C3—C5—C4	0.45 (17)		

### Hydrogen-bond geometry (Å, º)

**Table:**

*D*—H···*A*	*D*—H	H···*A*	*D*···*A*	*D*—H···*A*
N1—H1*A*···O2^i^	0.86	2.11	2.876 (2)	148
C7—H7*A*···O1^ii^	0.96	2.54	3.453 (5)	159

Symmetry codes: (i) *x*−1/2, −*y*+1/2, *z*+1/2; (ii) *x*+1/2, −*y*+1/2, *z*−1/2.
